# Cyclin D1 in Cancer: A Molecular Connection for Cell Cycle Control, Adhesion and Invasion in Tumor and Stroma

**DOI:** 10.3390/cells9122648

**Published:** 2020-12-09

**Authors:** Francesca Ida Montalto, Francesca De Amicis

**Affiliations:** 1 Department of Pharmacy, Health and Nutritional Sciences, University of Calabria, 87036 Rende, Italy; francescamontalto.93@libero.it; 2 Health Center, University of Calabria, 87036 Rende, Italy

**Keywords:** cell cycle, cyclin D1, breast cancer, oncogenic properties, tumor microenvironment

## Abstract

Cyclin D1, an important regulator of cell cycle, carries out a central role in the pathogenesis of cancer determining uncontrolled cellular proliferation. In normal cells, Cyclin D1 expression levels are strictly regulated, conversely, in cancer, its activity is intensified in various manners. Different studies demonstrate that *CCDN1* gene is amplified in several tumor types considering it as a negative prognostic marker of this pathology. Cyclin D1 is known for its role in the nucleus, but recent clinical studies associate the amount located in the cytoplasmic membrane with tumor invasion and metastasis. Cyclin D1 has also other functions: it governs the expression of specific miRNAs and it plays a crucial role in the tumor-stroma interactions potentiating most of the cancer hallmarks. In the present review, we will summarize the current scientific evidences that highlight the involvement of Cyclin D1 in the pathogenesis of different types of cancer, best of all in breast cancer. We will also focus on recent insights regarding the Cyclin D1 as molecular bridge between cell cycle control, adhesion, invasion, and tumor/stroma/immune-system interplay in cancer.

## 1. Introduction

Cyclin D1 is mostly known for the role played in the nucleus, as regulator of cell cycle progression. Cyclin D1 modulates the transition from G1 to S phase through its action as allosteric regulator of the cyclin-dependent kinase 4 (CDK) and CDK6. The active Cyclin D1/CDK4 complex translocates into the nucleus, phosphorylates retinoblastoma (RB) protein together with Cyclin E/CDK2, and dismisses the repressive action on the E2F transcription factor [1,2], which regulates the transcription of specific genes required for cell proliferation. The high expression of Cyclin D1 drives unchecked cellular proliferation promoting tumor growth, thus, the Cyclin D1 carries out a central role in the pathogenesis of cancer.

Together with the well-known role played by Cyclin D1 in cell cycle control, other functions are also described. Particularly, several studies investigate the effects of Cyclin D1 on mitochondrial metabolism [3,4], on the regulation of gene transcription [5,6]. It is reported that Cyclin D1 degradation is an essential component of the cellular response to genotoxic stress. [7].

Cyclin D1 is a 36-kDa protein encoded by *CCND1* gene, located on chromosome 11q13. Cyclin D1 is expressed by the majority of normal human cells apart from cells derived from bone marrow stem cell lines [8]. In numerous cells, Cyclin D1 is quite early induced by growth factors; next, it critically intercepts a number of intracellular pathways controlling cell proliferation. Dysregulation of Cyclin D1 transcription, accumulation and ubiquitination, as well as assembly and hyperactivation of its cognate CDK, lead to uncontrolled cell growth. Thus, Cyclin D1 is regarded as an oncogenic driver in different types of cancers including breast cancer, lung cancer, and melanoma [9]. The oncogenicity of Cyclin D1 was defined through in vivo models starting from studies of Bodrug et al., 1994, and Lovec et al., 1994 [10,11], which undoubtedly demonstrate that Cyclin D1 is involved in human B cell lymphoid malignancies, although they evidence that Cyclin D1 oncogenicity depends by collaboration with specific partners. Moreover, Wang et al. in 1994 [12] demonstrated that mice overexpressing Cyclin D1 in mammary epithelium display the predisposition to mammary cancer after a long latency period. Further data establish that Cyclin D1 is important for the development of mammary cancers induced by different oncoproteins such as receptor tyrosine-protein kinase erbB-2 (Her2/neu) [13] and v-Ha-ras, but not those induced by c-Myc or Wnt-1 [14]. These observations are consistent with data demonstrating that molecular signals targeted by Cyclin D1 overexpression cooperate with *p53* loss in breast cancer initiation. Notably, the development of several types of cancers is related to p53 inactivation together with Cyclin D1 dysregulation [15]. 

The role of Cyclin D1 in cancer initiation and progression appears complex and multifaceted, indicating that nowadays further investigations are needed for a more exhaustive comprehension of the therapeutic intervention targeting Cyclin D1-dependent mechanisms. Recent evidences strongly suggest the role of Cyclin D1 in the resistance to therapy and cancer progression. Cyclin D1 antagonizes BRCA1-mediated repression of estrogen receptor α (ERα)–dependent gene expression suggesting that silencing of *CCND1* combined with PARP inhibitors may lead to substantial benefit for several type of cancer patients [16]. In this concern, orthotopic ovarian cancer models respond better to olaparib treatment after knockdown of *CCND1* gene compared with olaparib treatment alone [17]. Besides, Cyclin D1 and the autophagic degradation machinery [18] are together linked to hepatocellular carcinoma (HCC) tumorigenesis [19]. It is reported that rise of autophagy-dependent Cyclin D1 degradation using amiodarone and rapamycin, represses tumor growth in both the orthotopic liver tumor and subcutaneous tumor xenograft models [19]. The proliferative status of the induced tumors is not significantly different regardless of the Cyclin D1 localization in the nucleus or in the cytoplasm.

Notably, an important role of cytoplasmic and membrane-associated Cyclin D1 for the regulation of cellular invasive capacity is emerged [20,21]. Based on the first major studies [20,22,23,24], more recently authors demonstrated that Cyclin D1 plays a role in the dissemination of glioblastoma (GBM) in vivo [25]. Using GBM xenografts, it is shown that invasive GBM cells, but not cells within the tumor masses, express a Cyclin D1 mostly located in the cytoplasm, and forced membrane accumulation of Cyclin D1 induces the number of invading cells. 

This review focuses on novel acquisitions regarding Cyclin D1 dysregulation in the cell cycle control, emerging from current research in human cancer. We first discuss the molecular basis underlying the role of Cyclin D1 in cancer initiation, progression and metastasis, and then, we highlight the role of Cyclin D1/CDK in the complex interplay between tumor and stroma. Furthermore, we discuss the preclinical rationale for targeting Cyclin D1/CDK in combination with current immunotherapy.

## 2. Cyclin D1-Mediated Mechanisms and Alterations in Human Cancer

Cyclin D1 protein consists of an RB protein binding portion, a domain for CDK binding or CDK inhibitor, a LxxLL motif recruiting coactivators, PEST site for Cyclin D1 degradation and a residual threonine, which controls nuclear export and protein stability [26]. 

### 2.1. CDK-Dependent and -Independent Mechanisms 

The main role of Cyclin D1 is CDK dependent to initiate RB phosphorylation, relieve histone deacetylases (HDAC)-binding, and finally allow access of Cyclin E/CDK2 complexes to their target [1,2]. RB hyperphosphorylation disrupts its association with E2F and allows transcriptional activation of the S-phase genes. These mechanisms of action are widely investigated, however, still collect many scientific interests. For instance, very recently, it is reported that in breast tumors inhibition of important cellular energy sensor such as mTOR Complex (TORC) 1/2 causes a decrease of Cyclin D1 protein, RB phosphorylation and E2F-mediated transcription. Combination of an mTORC1/2 inhibitor with a CDK4/6 inhibitor results in more profound effects on E2F-dependent transcription, which translate into a more durable growth arrest and a delay in the onset of therapy resistance [27].

Cyclin D1, through CDK-dependent and -independent mechanisms, can function as a transcriptional modulator by regulating the activity of several transcription factors involved at different levels in the cell cycle control, such as: (1) phosphorylation and inhibition of mediators of the transforming growth factor beta (TGF-β) such as Smad3 and consequent release of G1 arrest [28]; (2) activation of forkhead Box M1 (FOXM1) transcriptional function, control of several factors of G1/S phase, decrease of reactive oxygen species (ROS), inhibition of cancer cells senescence [29]; (3) association with histone acetyltransferase (HAT) complexes, such as P300/CBP-associated factor (P/CAF) to regulate both ERα and androgen receptor (AR) activity in breast and prostate epithelial cells [30,31]. Notably, Cyclin D1 regulates cell cycle, cell growth, and cell differentiation in CDK-independent manner, by binding nuclear receptors such as ERα, AR, peroxisome proliferator-activated receptor (PPAR) γ, thyroid hormone receptor beta (TR-β), and multiple nuclear receptor coregulators [32]. For instance, Cyclin D1 can directly bind to the hormone-binding domain of ERα, mediating gene transcription also in the absence of estrogens. Thus, Cyclin D1 may be retained as an independent activator of ERα [33]. Remarkably, data show the physical interaction between Cyclin D1 and ERα, paralleled by increases in ligand-independent ER-mediated transcription from an estrogen response element containing reporter construct. The level of transactivation indicates that maximal activation of ERα can be achieved by Cyclin D1 overexpression. This mechanism may explain how Cyclin D1 contributes to ERα activation [32] driving breast cancer initiation and progression. Therefore, both increased expression of Cyclin D1 and ERα positivity are valuable indicators for the prediction of recurrence and prognosis for breast cancer patients [34].

Conversely, Cyclin D1 attenuates the transactivation potential of the AR and it is reported that Cyclin D1 modulates the androgen-dependent molecular profile [35]. In prostate cancer AR target genes are suppressed by Cyclin D1, which limits, in such a way, the AR activity induced by its specific ligands. Moreover, Cyclin D1 markedly reduces the AR recruitment on clinically relevant gene loci. Thus, these studies identify the transcriptional regulatory functions of Cyclin D1 as critical effector of androgen-dependent signaling and AR-associated chromatin dynamics [36].

Cyclin D1 is also a corepressor for thyroid hormone receptors (TR) [37]. During transcription, Cyclin D1 acts as a bridging factor to recruit HDAC3; this causes the silencing activity of the unbound TR and the repression of the thyroid hormone-dependent transcriptional activity [37]. Therefore, the repression of TR transcriptional activity, mediated by Cyclin D1, generates a negative feedback loop to maintain the regulatory network controlling the expression of target genes involved in cell cycle control.

Several studies demonstrate the regulation of PPARs signaling by Cyclin D1 [38]. It is likely that Cyclin D1 plays a dual role by promoting cell proliferation and inhibiting cellular differentiation. Analysis of tissues and cells from Cyclin D1^–/–^ mice demonstrate an important physiological role for Cyclin D1 as an inhibitor of adipocyte differentiation through repression of PPARγ function [38]. Further studies indicate that Cyclin D1 inhibits the activity of PPARα, a key metabolic transcription factor, that induces fatty acid oxidation. In primary hepatocytes, Cyclin D1 inhibits PPARα transcriptional activity and gene expression in a CDK4-independent manner. In liver and breast cancer cells, knockdown of *CCND1* gene leads to increased PPARα transcriptional activity, expression of PPARα target genes, and fatty acid oxidation [39]. Thus, these data highlight a possible molecular link between Cyclin D1 and cellular metabolism influencing cancer cells proliferation.

### 2.2. Amplification or Overexpression of Cyclin D1

During G1 phase, nuclear import controls the amount of Cyclin D1/CDK4 complexes in the nucleus; next, during S-phase, Cyclin D1 becomes phosphorylated and exported from the nucleus [40]. Nuclear export is coupled with ubiquitin-dependent destruction of Cyclin D1 in the cytoplasm. Thus, in normal cells, Cyclin D1 expression levels are strictly regulated. In contrast, many carcinomas are characterized by Cyclin D1 overexpression [41]. For instance, the amplification of the *CCDN1* gene sequence or overexpression of Cyclin D1 result in dysregulated CDK activity, alteration of cell cycle control, bypass of key cellular checkpoints, induced cell proliferation, and ultimately neoplastic growth [42].

The Cyclin D1 is overexpressed in up to 50% of breast cancer, and amplification of the *CCND1* gene is associated with a poor patient outcome in several studies [41]. In breast tumors, higher Cyclin D1 positive rate corresponds to higher ER positive rate but lower Her-2 positive rate [43]. Nevertheless, controversy regarding overexpression of Cyclin D1 protein and/or *CCDN1* gene amplification in relation to survival of cancer patients still exists.

For instance, it is reported that the amplification of *CCND1* contributes to the loss of cell cycle control only in a small fraction of malignant gliomas [44]. In an interesting study Lundgren et al. characterizes the association between *CCND1* amplification, Cyclin D1 protein levels, and breast cancer recurrence, in a large randomized cohort of ER-positive breast cancer postmenopausal patients, treated with endocrine therapy. Clinical data indicate that amplification of *CCND1* observed in 8.7% of the tumors associates with increased risk of disease recurrence. In contrast, both higher nuclear expression and high cytoplasmic expression of Cyclin D1 instead associate with a decreased recurrence risk. No difference is observed in the response to different therapies with aromatase inhibitors (anastrozole) compared with tamoxifen, according to *CCDN1* gene amplification or protein expression [45]. The amplification of *CCDN1* and deletions of *PPP2R2A* gene, encoding for a serine/threonine-protein phosphatase marker of worse prognosis, have been recently associated to a subgroup of luminal breast carcinoma that exhibits poor survival. The combined analysis of Cyclin D1 and PPP2R2A expression, assessed by immunohistochemistry, identified an immunophenotype of luminal breast that associates with poor overall survival and disease-free survival [46]. Significant copy number alterations of *CCND1* and *EGFR* correlate with clinical stage, tumor differentiation, and lymph node metastasis in oral squamous cell carcinoma (OSCC). Furthermore, *CCND1* and *EGFR* show an additive effect on OSCC tumor progression [47]. Exome sequencing analysis of 243 liver tumors demonstrates mutational signatures associated with specific risk. *CCND1* amplification, together with 161 putative driver genes and *p53* alterations, is evidenced at more advanced stages in aggressive tumors [48].

## 3. Cyclin D1: Role as Cell Cycle Regulator in Tumor Behavior

### 3.1. Cyclin D1 in Breast Cancer 

**Figure 1:**
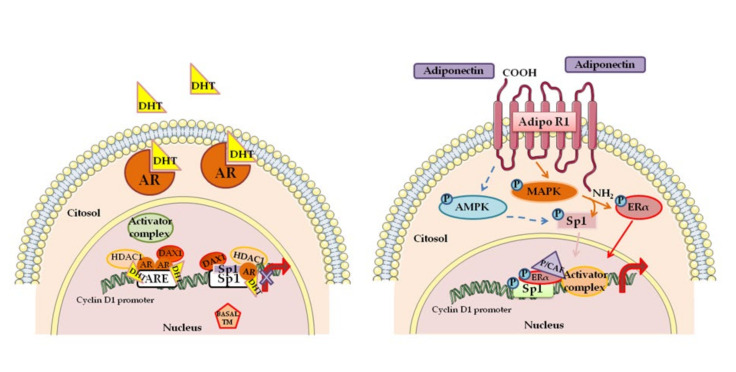
A schematic summary illustrating mechanisms targeting Cyclin D1 in estrogen receptor α (Erα)-positive breast cancer cells. LEFT PANEL Cyclin D1 as a key target of dihydrotestosterone (DHT)/androgen receptor (AR) action in breast cancer cells for the repression of cell proliferation [54]. RIGHT PANEL. Cyclin D1 as a key target of adiponectin action in ERα- positive breast cancer cells. Adiponectin induced signals, AMP-activated protein kinase (AMPK) and mitogen-activated protein kinase (MAPK), phosphorylate both specificity protein 1 (Sp1) and ERα, producing the recruitment of an activator complex on Cyclin D1 promoter. This causes the increased Cyclin D1 expression and breast tumor growth [55]. See the text for details. Abbreviations: DAX1 (corepressor complex component), ARE (androgen responsive element), histone deacetylases (HDAC1), Basal TM (basal transcriptional machinery) Adipo R1 (Adiponectin receptor 1), P/CAF (P300/CBP-associated factor).

In hormone-dependent breast carcinoma, estradiol and ERα regulate initiation and progression through transcriptional activation of specific genes. In this concern, the induction of Cyclin D1 expression and/or the activation of multiple simultaneous pathways converging on the Cyclin D1-CDK4/6 axis are fundamental [49]. Interestingly, combination of antiestrogen therapies, including tamoxifen and CDK inhibitors, induce cell cycle arrest in the G1 phase [50]. Not only ERs but also other receptors such us progesterone receptor (PR), human Her2, and AR play important prognostic and predictive roles in the pathogenesis of breast cancer. Androgens, through their own receptor AR, exert a protective role on breast tumor development and progression, counteracting the estrogen activity [51,52,53]. Cyclin D1 expression is negatively modulated by dihydrotestosterone (DHT) through a genomic mechanism dependent by AR (See Figure 1 left panel).

The latter is recruited at the androgen responsive element (ARE)- and specificity protein 1 (Sp1)-containing region of the proximal *CCDN1* gene promoter, with consequent suppression of Cyclin D1 transcription [54]. These data support the idea that inhibition Cyclin D1 expression by AR may be a protective mechanism to reduce estradiol-dependent cell cycle progression in breast cancer cells [56]. Modulation of Cyclin D1 levels accordingly to ERα expression could explain the double action of the adipokine adiponectin in breast cancer occurrence and progression [57]. In animals injected with human ERα-negative breast cancer cells, pretreated with adiponectin, authors observed a reduction of tumor volume, whereas in the animals receiving human ERα-positive cells, authors observed an increase of tumor growth. Further in vitro experiments demonstrate opposite effects of adiponectin on Cyclin D1 expression levels and gene promoter activity. In ERα-positive cells, the adiponectin produces the specific recruitment of a coactivator complex inducing Cyclin D1 transcription, sustaining ERα-positive breast cancer cell proliferation. (See Figure 1 right panel). In ERα-negative breast cancer cells, upon adiponectin treatment, Sp1 is phosphorylated by AMP-activated protein kinase (AMPK) and recruited on the responsive region of the Cyclin D1 promoter, causing the recruitment of a corepressor complex, the displacement of RNA Polomerase II, and the reduction Cyclin D1 transcriptional activity [55]. 

Recent studies show a specific role of short, noncoding RNA (miRNAs) in breast cancer pathogenesis [58]. These findings open new therapeutic perspectives and a new era of biomedical science. However, despite showing immense promise, miRNAs have not been successfully implemented in the clinical setting due to a lack of a standardized approach and conflicting results [59]. In addition to regulating the coding region, Cyclin D1 has been shown to regulate noncoding miRNA and vice versa. Several studies focus on the human miR-17/20 cluster, whose amplification and overexpression has been described in different type of cancers [60]. Notably, authors demonstrate that miR17/20 inhibits breast cancer cellular proliferation by repression of Cyclin D1 translation, via a conserved 3’ untranslated region miRNA-binding site. Furthermore, the cell cycle effect of miR-17/20 disappears after Cyclin D1 silencing and in Cyclin D1-deficient breast cancer cells. Besides, in the Cyclin D1-induced mammary tumors, the miR-17/20 cluster appears upregulated, since Cyclin D1 is able to bind the miR-17/20 cluster promoter regulatory region [61]. The mechanism by which Cyclin D1 regulates noncoding RNA is further defined by studies of miRNA processing. Cyclin D1^−/−^ cells are characterized by reduction in pre-miRNA processing, which is restored by Cyclin D1 rescue. Indeed, in vitro and in vivo experiments demonstrate that Cyclin D1 induces the expression of RNase III endoribonuclease Dicer, which cleaves long double-stranded RNA (dsRNA) or stemloop-stem-structured pre-miRNA to form mature miRNAs. Particularly, Dicer is transcriptionally modulated by Cyclin D1, through a CDK-independent mechanism. Since Cyclin D1 and Dicer expressions significantly correlate in luminal A and basal-like subtypes of human breast cancer, authors conclude that Cyclin D1 through Dicer coordinates microRNA biogenesis in these breast cancer subtypes [62]. Further elegant studies demonstrate that Cyclin D1 governs a specific cluster of miRNA, which correlates with ERα positivity in breast cancer and with Wnt signaling activation. The Cyclin D1-mediated miRNA signature engages a network of coding genes, including the Dickkopf Wnt signaling pathway inhibitor (DKK) proteins antagonists of the Wnt signaling. Thus, Cyclin D1 governs an additional regulatory mechanism that activates Wnt via non-coding miRNA [63]. Interestingly, further recent data report that Cyclin D1 induces the secretion of specific miRNAs governing the tumor immune response. miR-21 and miR-93, which bind Toll-Like Receptor 8 to trigger a pro-metastatic inflammatory response, represent >85% of the Cyclin D1-induced secreted miRNA transcripts. Thus, these researches, demonstrate an unknown function of Cyclin D1, suggesting increased secretion of immuno-miRNAs that lead a pro-tumorigenic immune phenotype [64]. 

### 3.2. Cyclin D1 in Hepatocellular Carcinoma

A new role for Cyclin D1 in controlling liver cell proliferation and cancer stem cells self-renewal is rapidly emerging. During hepatic carcinogenesis, Cyclin D1 is induced by activated β-catenin, it can partially substitute activated β-catenin and cooperates with the oncogene *MET* to induce liver cancer formation in vivo [65]. Besides, Cyclin D1 modulates liver cancer stem cells self-renewal, interacting with and activating Smad2/3 and Smad4. Accordingly, the Cyclin D1-dependent activation of Smad2/3 and Smad4 is also evidenced in hepatocellular carcinoma (HCC) patients and predicts disease progression [66]. Increased expression of the Cyclin D1 mRNA is observed in patients with HBV-HCC [67], however, further in vivo models show that over-expression of Cyclin D1 alone may not be sufficient for HCC development. Recent evidences indicate that a high expression of Minichromosome maintenance complex component (MCM) 7 is associated with poor prognosis in HCC patients. The MCM7 plays an essential role in initiating DNA replication and its upregulation increases the proliferation of HCC cells in vitro and tumorigenicity in vivo. The combination of MCM7 and Cyclin D1 has a prognostic value in HCC patients. Indeed, a positive association between MCM7 and Cyclin D1 expression is found in mouse models and human tumor tissues. DNA damage and chromosomal abnormalities cause the overexpression of Cyclin D1 and its accumulation in cancer cell nuclei interfering in cell cycle control, further suggesting the crucial role of the nuclear Cyclin D1 in this type of cancer [68]. 

### 3.3. Cyclin D1 in Ovary Cancer

Ovarian cancer (OC) is a significant cause of mortality, because patients develop chemoresistance after initial response to chemotherapy [69]. Several studies demonstrate a role of Cyclin D1 in initiation and progression of the disease. Chen et al., 2015 discovered that follicle-stimulating hormone (FSH) stimulates proliferation of epithelial OC, by regulation of Cyclin D1 expression. FSH-induced Cyclin D1 expression is mediated by the candidate oncogene gankyrin, usually expressed at higher levels [70,71].

Important evidences obtained by the large-scale cancer genomic analysis indicate that the genes of cell cycle control are altered in 70% of high-grade OC [72]. Specifically, the overexpression of Cyclin D1 is often detected, and it is strongly associated with chemoresistance and poor prognosis in OC, thus, Cyclin D1 overexpression significantly influences the clinical outcome in advanced epithelial OC [73]. Similarly, Bali et al. identified Cyclin D1 overexpression as an independent prognostic factor in the multivariate analysis of 134 serous OC cases [74]. A database search evidences that in human OC tissues miR-211 is significantly downregulated in clear-cell, an uncommon, but aggressive epithelial OC and high-grade serous carcinomas, compared to healthy control tissue. Authors demonstrate that miR-211 directly binds to sequences in Cyclin D1 and CDK6 mRNA, repressing their translation into protein [75]. 

### 3.4. Cyclin D1 in Lung Cancer 

Cyclin D1 overexpression is reported in 18–76% of evaluated patients [76] and *CCND1* gene amplification in 5–32% of non-small cell lung carcinoma patients (NSCLC). Oncogenic Cyclin D1 overexpression produces a global transcriptome downmodulation probably due to the interaction of Cyclin D1 with the transcription machinery [77]. Moreover, KRAS-driven lung tumorigenesis requires the activation of extracellular signal-regulated kinase (ERK)/Cyclin D1 pathway in human-lung adenocarcinoma tissues and transgenic mice [78]. These studies suggest a possible cooperation of Cyclin D1 with KRAS in early pathogenesis of NSCLC. Similarly to KRAS, also the alteration of the oncosuppressor *phosphatase and tensin homolog* (*PTEN*) [79] is associated with the overexpression of Cyclin D1. Different studies show that wild-type PTEN prevents the increase in nuclear localization of Cyclin D1 and induces G1 cell cycle arrest. Not only in NSCLC but also in other types of cancer the depletion of PTEN is significantly correlated with increased Cyclin D1 expression [80]. In patients with NSCLC, it has been observed also that hypermethylation of *PTEN* [81], *EGFR* gene’s amplification, and oncogene *MET* coexistence are correlated with Cyclin D1 overexpression [82].

## 4. Membrane Associated and Cytoplasmatic Cyclin D1: Linking Cell Cycle Control, Adhesion and Invasion

Cyclin D1 is especially known for its catalytic and noncatalytic roles regarding the transcriptional regulation of genes that are involved in proliferation and differentiation. However, recent data demonstrate that Cyclin D1 complexes also target substrates with roles in cytoskeletal modelling, cell adhesion, and motility [20]. Particularly, Cyclin D1 is not restricted to the nucleus but it is also associated to the cytoplasm and cytoplasmic membrane, where it can activate cytoplasmic targets involved in cell invasive potential [21].

Pioneering evidences demonstrate that Cyclin D1 deficiency in bone marrow-derived macrophages increases focal complex formation and adhesion, yielding a flattened, circular morphology with reduced membrane ruffles [20]. Moreover, Cyclin D1 loss is associated with reduced cellular migration in response to different stimuli. Besides, Cyclin D1-loss in mouse embryo fibroblasts (MEFs) and mammary epithelial cells causes increase of cell adhesion and decrease of motility compared with wild-type cells [23,24]. This phenotype is counteracted by transduction of Cyclin D1^−/−^ cells with a Cyclin D1 cDNA, inducing cell migration and reduction of the activity of Rho GTPase, master of cellular migration, governing spatiotemporal activation and coordination of subsequent protein–protein and protein–lipid interactions [83].

**Figure 2:**
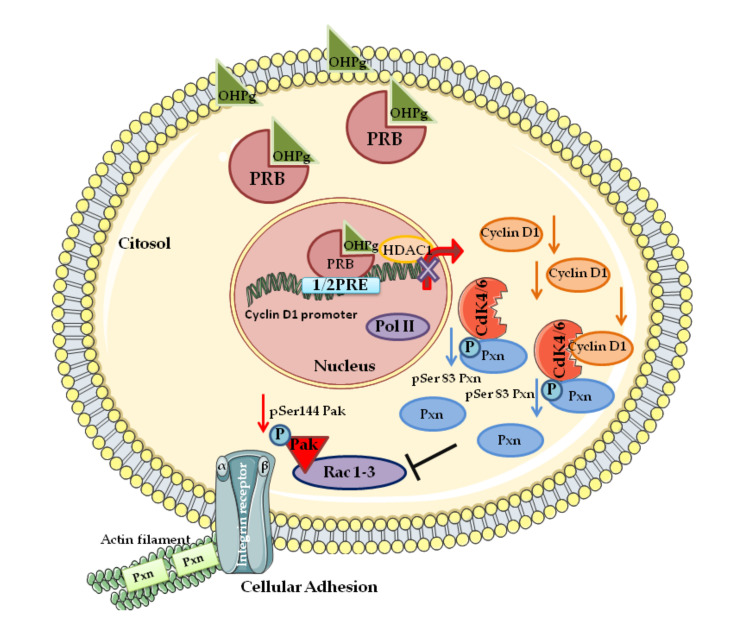
A schematic summary illustrating Cyclin D1 as a key target of ligand-activated progesterone receptor (PR)-B in breast cancer cells invasion. [88]. hydroxyprogesterone (OHPg)/PR-B reduce Cyclin D1 expression by transcriptional regulation due to the recruitment of PR-B on Cyclin D1 promoter. Cyclin D1/cyclin-dependent kinase 4 (CDK4)/paxillin (Pxn) complex, pSer83Pxn, Rac, and Pak are inhibited causing delay of cell invasion and migration. See the text for details. Abbreviations: PRE (progesterone responsive element), histone deacetylases (HDAC1), Pol II (RNA polymerase II).

Further studies identify Cyclin D1 in the cytoplasmic membrane [84] and show Cyclin D1 to interact and modulate the function of several cytoplasmic membrane-associated proteins including the filamin A [85], protein kinase C, casein kinase substrate in neurons protein 2 (PACSIN II) [22], and paxillin (Pxn) [86]. Paxillin, a key component of focal adhesion, interacts with many molecules thus monitoring the Rho GTPases [87]. Notably, Pxn contains many phosphorylation sites, and it was demonstrated that Pxn acts as a substrate for the Cyclin D1/CDK4 complex [86]. We recently reported that low levels of Pxn phosphorylation at Ser 83, resulting to the reduced Cyclin D1/CDK4 functional interaction, led to the inhibition of mediators of membrane ruffles and actin rearrangements such as Rac1 and its effector Pak, as evidenced by the reduction of pSer144-Pak1 levels [88] (see Figure 2). 

High levels of Cyclin D1/CDK4 also promote GBM cells dissemination. Cyclin D1 induces the invasion of primary human GBM cells through a cytoplasmic RB protein-independent mechanism. Using GBM mouse models, authors discovered that evaded GBM cells (such as perivascular cells) show that Cyclin D1 colocalizes with regulators of cell invasion such as RalA and Pxn. Genetic data indicate that, in GBM cells, the Cyclin D1/CDK4 complex is acting upstream of those regulators. Hence, expression of Cyclin D1 induces focal adhesion kinase, RalA and Rac1 activities. In agreement with these findings in vivo experiments demonstrated increased GBM dissemination after exogenous expression of membrane-targeted Cyclin D1 [25]. 

Analyzing by immunohistochemistry of the localization of Cyclin D1 in endometrial, breast, prostate, and colon carcinomas with different types of invasion, Fustè et al. [89] found that in prostatic adenocarcinoma Cyclin D1 displays an asymmetric pattern of localization. Specifically, cytoplasmic-membranous Cyclin D1 expression correlates with the Gleason grade and, the higher expression is evidenced in pT3, that is, when tumor extends beyond the prostate [89]. In the cells exogenously expressing a membrane-attached variant of Cyclin D1, authors observed that the sequestration of Cyclin D1 in the membrane enhances cell invasion and metastasis without affecting cell proliferation.

Future studies could be aimed at characterizing the partners of Cyclin D1 responsible for these noncanonical oncogenic features, using proteomic screen in Cyclin D1-expressing cell models [90]. Body et al. demonstrate that Cyclin D1 accumulates in the cytoplasm in mantle cell lymphoma (MCL) cell lines and primary tumors. They investigate the main molecular functions of Cyclin D1 in MCL, by identifying factors interacting with Cyclin D1. Proteomic data reveal a large number of Cyclin D1-interacting factors involved in cell migration, invasion, and adhesion [91]. Most of these partners of Cyclin D1 found in B-cell lymphoma and myeloma cells, are also present in solid tumors such as breast cancer, squamous cell carcinoma, and colorectal cancer. Although further studies are required to address the specific role mediated by Cyclin D1 in the cytoplasm, at least several of the above-described effects on cancer cell migration/invasion might be targets for the development of improved combinatorial cancer treatment regimens. Indeed, all these data suggest that pharmacological inactivation of Cyclin D1/CDK could be included in the therapeutic portfolio, not only to counteract tumor cell proliferation but also against the invasive capacity.

## 5. Cyclin D1 Role in the Tumor Microenvironment 

The tumoral microenvironment is an essential aspect of the tumor, since it provides a nurturing for the malignant process. Particularly, the relationship between the two components of the solid tumors, tumor cells, and stromal cells is essential for tumor growth, progression, and development of metastasis [92]. Stromal cell population in many tumor types is characterized by cancer-associated fibroblasts (CAFs), cells from different cell populations including bone-marrow mesenchymal stem cells, epithelial to mesenchymal transition cancer cells, endothelial cells, and resident fibroblast [93]. Understanding the molecular interaction between tumor cells and stroma cells provides new and valuable clinical targets for cancer treatment. A growing scientific interest is directed to the role of Cyclin D1 in this complex interplay and how it is correlated with proliferative signaling, cellular energetics, inflammation, angiogenesis, activating invasion, and metastasis all contributing with tumor progression. A pioneering study evidences the importance of Cyclin D1 as a crucial regulator of tumor-stroma interactions, in prostate cancer progression [94]. By exogenous expression of Cyclin D1 in benign stromal cells, authors found that cell behavior mimics that of cancer stromal cells. Particularly, the Cyclin D1-overexpressing fibroblasts have an increased life span and share several similarities with CAFs. Microarray studies demonstrate a high concordant gene expression profile between the Cyclin D1-overexpressing fibroblasts and CAFs, compared with normal prostate fibroblasts. Subsequent in vivo studies establish that the Cyclin D1/CDK4 axis plays a critical role not only in glial tumor cells but also in stromal-derived cells, which sustain tumor growth [95]. Specifically, tumor-associated microglia (TAM) in tumors from Cyclin D1 and CDK4 knockout mice show a cell morphology characterized by multiple protrusions associated with a reduced state of activation. Cathepsin and other markers of TAM activation are reduced. Moreover, platelet-derived growth factor–transformed glial cells engrafted orthotopically into the mice, formed more aggressive tumors in wild-type mice compared with Cyclin D1-deficient animals. This in vivo model suggests the idea that loss of Cyclin D1 in TAMs impedes the progression to increased malignancy. Thus, therapies directed to Cyclin D1/CDK4 may be useful in targeting both the tumor cell and modulating the activity of a stromal cell type that is critical to support malignant progression.

More recent studies show that the expression of the Cyclin D1 increases in human cancer stroma, promoting tumor inflammation, angiogenesis, and stem cell expansion in advanced breast cancer. An increase of nuclear Cyclin D1 levels in stromal fibroblast of 914 breast cancer patients has been demonstrated, predicting poor outcome. This increase correlates with the secretion of factors that promote expansion of stem cells, such as breast stem-like cells, embryonic stem cells, and bone marrow-derived stem cells, and induces an inflammatory infiltration increasing the recruitment of F4/80+ and CD11b+ macrophages and angiogenesis. Stromal Cyclin D1 also increases the secretion of pro-inflammatory cytokines (CCL2, CCL7, CCL11, CXCL1, CXCL5, CXCL9, CXCL12), CSF (CSF1, GM-CSF1) and osteopontin (OPN) [96]. These data demonstrate that Cyclin D1 expression in stroma cells regulates tumor microenvironment development, promoting several key hallmarks of cancer.

A very recent database investigation suggests that *CCND1* amplification may be associated with poor clinical response to antiestrogen therapy in breast cancer patients, through suppressing the antitumor immunity in TME [97]. Transcriptomic analysis shows various degrees of immune cell exclusion in the population characterized by *CCND1* amplification. The gene set enrichment analysis indicates that *CCND1* amplification correlates with multiple aggressive, immunosuppressive hallmarks including epithelial-mesenchymal transition and hypoxia signaling. Some limitations to these conclusions are related to other issues such as tumor type, standardization of detection, and accompanying gene mutation status. Therefore, the full implication of Cyclin D1 in the molecular connection between tumor and stroma deserves more complete investigation regarding the mechanism of *CCND1* amplification and primary immune resistance.

## 6. Conclusions

Cyclin D1 through its binding partners CDKs is a key regulator of the cell cycle and is overexpressed with high frequency in many human cancers. A series of studies demonstrate how Cyclin D1 is essential in the mechanisms involved in the pathogenesis of cancer. In recent years, in vitro and in vivo studies have delineated a new position of Cyclin D1 as controller of cellular invasiveness and aggressiveness. Notably, Cyclin D1 overexpression is a fundamental determinant of the reciprocal interplay between cancer cells and the stroma, exerting in such a way a “tumor-promoting” action. All these concepts support the use of CDKs inhibitors in cancer therapy. Notably, CDK4/6 inhibitors are small selective, orally bioavailable molecules able to restrain proliferation of sensitive cancer cells, preventing cell cycle progression. The recent discovery of ribociclib and abemaciclib, added to the therapeutic portfolio including palbociclib, contributes to increase the response rates and prolongs disease control when combined with endocrine therapy in hormone-responsive advanced breast cancer. Moreover, a number of preclinical studies indicate that these molecules are able to determine an antitumor immune response, evidenced by the combination with immune checkpoint blockade, targeting pathways such as the programmed cell death protein-1 (PD-1) axis resulting in a synergistic effect on tumor growth [98]. These promising results of durable responses in patients with advanced cancer also underscore the importance of understanding the roles of the Cyclin D1 in shaping the development of the tumor microenvironment and in the therapeutic efficacy for successful clinical translation in treating patients with cancer.

Assessing the contribution of each function of Cyclin D1 to cancer progression could contribute to develop therapies in an appropriately targeted and personalized manner. 

## References

[1] Kato J., Matsushime H., Hiebert S.W., Ewen M.E., Sherr C.J. (1993). Direct binding of cyclin D to the retinoblastoma gene product (pRb) and pRb phosphorylation by the cyclin D-dependent kinase CDK4. Genes Dev..

[2] Lundberg A.S., Weinberg R.A. (1998). Functional Inactivation of the Retinoblastoma Protein Requires Sequential Modification by at Least Two Distinct Cyclin-cdk Complexes. Mol. Cell. Biol..

[3] Wang C., Li Z., Lu Y., Du R., Katiyar S., Yang J., Fu M., Leader J.E., Quong A., Phyllis M. (2006). Cyclin D1 repression of nuclear respiratory factor 1 integrates nuclear DNA synthesis and mitochondrial function. Proc. Natl. Acad. Sci. USA.

[4] Sakamaki T., Casimiro M.C., Ju X., Quong A.A., Katiyar S., Liu M., Jiao X., Li A., Zhang X., Lu Y. (2006). Cyclin D1 Determines Mitochondrial Function In Vivo. Mol. Cell. Biol..

[5] Hulit J., Bash T., Fu M., Galbiati F., Albanese C., Sage D.R., Schlegel A., Zhurinsky J., Shtutman M., Ben-Ze’ev A. (2000). The Cyclin D1 Gene Is Transcriptionally Repressed by Caveolin-1. J. Biol. Chem..

[6] Bienvenu F., Jirawatnotai S., Elias J.E., Meyer C.A., Mizeracka K., Marson A., Frampton G.M., Cole M.F., Odom D.T., Odajima J. (2010). Transcriptional role of cyclin D1 in development revealed by a genetic–proteomic screen. Nature.

[7] Agami R., Bernards R. (2000). Distinct Initiation and Maintenance Mechanisms Cooperate to Induce G1 Cell Cycle Arrestin Response to DNA Damage. Cell.

[8] Inaba T., Matsushime H., Valentine M., Roussel M.F., Sherr C.J., Look A.T. (1992). Genomic Organization, Chromosomal Localization, and Independent Expression of Human Cyclin D Genes. Genomics.

[9] Tchakarskaa G., Sola B. (2020). The double dealing of cyclin D1. Cell Cycle.

[10] Bodrug S.E., Warner B.J., Bath M.L., Lindeman G.J., Harris A.W., Adams J.M. (1994). Cyclin D1 transgene impedes lymphocyte maturation and collaborates in lymphomagenesis with the myc gene. EMBO J..

[11] Lovec H., Grzeschiczek A., Kowalski M.B., Moroy T. (1994). Cyclin D1/bcl-1 cooperates with myc genes in the generation of B-cell lymphoma in transgenic mice. EMBO J..

[12] Wang T.C., Cardiff R.D., Zukerberg L., Lees E., Arnold A., Schmidt E.V. (1994). Mammary hyperplasia and carcinoma in MMTV-cyclin D1 transgenic mice. Nature.

[13] Lee R.J., Albanese C., Fu M., D’Amico M., Lin B., Watanabe G., Haines G.K., Siegel P.M., Hung M.C., Yarden Y. (2000). Cyclin D1 is required for transformation by activated Neu and is induced through an E2F-dependent signaling pathway. Mol. Cell. Biol..

[14] Yu Q., Geng Y., Sicinski P. (2001). Specific protection against breast cancers by cyclin D1 ablation. Nature.

[15] Hosokawa Y., Papanikolaou A., Cardiff R.D., Yoshimoto K., Bernstein M., Wang T.C., Schmidt E.V., Arnold A. (2001). In vivo analysis of mammary and non-mammary tumorigenesis in MMTV-cyclin D1 transgenic mice deficient in p53. Transgenic Res..

[16] Wang C., Fan S., Li Z., Fu M., Rao M., Ma Y., Lisanti M.P., Albanese C., Katzenellenbogen B.S., Kushner P.J. (2005). Cyclin D1 Antagonizes BRCA1 Repression of Estrogen Receptor α Activity. Cancer Res..

[17] Zhong Q., Hu Z., Li Q., Yi T., Li J., Yang H. (2019). Cyclin D1 silencing impairs DNA double strand break repair, sensitizes BRCA1 wildtype ovarian cancer cells to olaparib. Gynecol. Oncol..

[18] Casimiro M.C., Di Sante G., Di Rocco A., Loro E., Pupo C., Pestell T.G., Bisetto S., Velasco-Vel_azquez M.A., Jiao X., Li Z. (2017). Cyclin D1 Restrains Oncogene-Induced Autophagy by Regulating the AMPK–LKB1 Signaling Axis. Cancer Res..

[19] Wu S.-Y., Lan S.-H., Wu S.-R., Chiu Y.-C., Lin X.-Z., Su I.-J., Tsai T.-F., Yen C.-J., Lu T.-H., Liang F.-W. (2018). Hepatocellular Carcinoma–Related Cyclin D1 Is Selectively Regulated by Autophagy Degradation System. Hepatology.

[20] Neumeister P., Pixley F.J., Xiong Y., Xie H., Wu K., Ashton A., Cammer M., Chan A., Symons M., Stanley E.R. (2003). Cyclin D1 governs adhesion and motility of macrophages. Mol. Biol. Cell.

[21] Chen K., Jiao X., Ashton A., Di Rocco A., Pestell T.G., Sun Y., Zhao J., Casimiro M.C., Li Z., Lisanti M.P. (2020). The membrane-associated form of cyclin D1 enhances cellular invasion. Oncogenesis.

[22] Meng H., Tian L., Zhou J., Li Z., Jiao X., Li W.W., Plomann M., Xu Z., Lisanti M.P., Wang C. (2011). PACSIN 2 represses cellular migration through direct association with cyclin D1 but not its alternate splice form cyclin D1b. Cell Cycle.

[23] Li Z., Wang C., Jiao X., Lu Y., Fu M., Quong A.A., Dye C., Yang J., Dai M., Ju X. (2006). Cyclin D1 regulates cellular migration through the inhibition of thrombospondin 1 and ROCK signaling. Mol. Cell. Biol..

[24] Li Z., Wang C., Prendergast G.C., Pestell R.G. (2006). Cyclin D1 functions in cell migration. Cell Cycle.

[25] Cemeli T., Guasch-Vallés M., Nàger M., Felip I., Cambray S., Santacana M., Gatius S., Pedraza N., Dolcet X., Ferrezuelo F. (2019). Cytoplasmic cyclin D1 regulates glioblastoma dissemination. J. Pathol..

[26] Musgrove E.A., Caldon C.E., Barraclough J., Stone A., Sutherlan R.L. (2011). Cyclin D as a therapeutic target in cancer. Nat. Rev. Cancer.

[27] Michaloglou C., Crafter C., Siersbaek R., Delpuech O., Curwen J.O., Carnevalli L.S., Staniszewska A.D., Polanska U.M., Cheraghchi-Bashi A., Lawson M. (2018). Combined Inhibition of mTOR and CDK4/6 Is Required for Optimal Blockade of E2F Function and Long-term Growth Inhibition in Estrogen Receptor–positive Breast Cancer. Mol. Cancer Ther..

[28] Zelivianski S., Cooley A., Kall R., Jeruss J.S. (2010). Cyclin-Dependent Kinase 4–Mediated Phosphorylation Inhibits Smad3 Activity in Cyclin D–Overexpressing Breast Cancer Cells. Mol. Cancer Res..

[29] Anders L., Kel N., Hydbring P., Choi Y.J., Widlund H.R., Chick J.M., Zhai H., Vidal M., Gygi S.P., Braun S. (2011). A Systematic Screen for CDK4/6 Substrates Links FOXM1 Phosphorylation to Senescence Suppression in Cancer Cells. Cancer Cell.

[30] McMahon C., Tuangporn S., Di Renzo J., Ewen M.E. (1999). P/CAF associates with cyclin D1 and potentiates its activation of the estrogen receptor. Proc. Natl. Acad. Sci. USA.

[31] Reutens A.T., Fu M., Wang C., Albanese C., McPhaul M.J., Sun Z., Balk S.P., Jänne O.A., Palvimo J.J., Pestell R.G. (2001). Cyclin D1 Binds the Androgen Receptor and Regulates Hormone-Dependent Signaling in a p300/CBP-Associated Factor (P/CAF)-Dependent Manner. Mol. Endocrinol..

[32] Inoue K., Fry E.A. (2015). Aberrant expression of cyclin D1 in cancer. Signal Transduct. Insights.

[33] Luo J., Chen Y., Xu H. (2010). Expression of B-catenin, cyclinD1 and Erα in breast cancer tissues. Prev. Med..

[34] Lamb J., Ladha M.H., Mcmahon C., Sutherland R.L., Ewen M.E. (2000). Regulation of the Functional Interaction between Cyclin D1 and the Estrogen Receptor. Mol. Cell. Biol..

[35] Knudsen K.E., Cavenee W.K., Arden K.C. (1999). D-Type Cyclins Complex with the Androgen Receptor and Inhibit Its Transcriptional Transactivation Ability. Cancer Res..

[36] Comstock C.E.S., Augello M.A., Schiewer M.J., Karch J., Burd C.J., Ertel A., Knudsen E.S., Jessen W.J., Aronow B.J., Knudsen K.E. (2011). Cyclin D1 Is a Selective Modifier of Androgen-dependent Signaling and Androgen Receptor Function. J. Biol. Chem..

[37] Lin H.M., Zhao L., Cheng S.Y. (2002). Cyclin D1 Is a Ligand-independent Co-repressor for Thyroid Hormone Receptors. J. Biol. Chem..

[38] Fu M., Rao M., Bouras T., Wang C., Wu K., Zhang X., Li Z., Yao T., Pestell R.G. (2005). Cyclin D1 inhibits peroxisome proliferator-activated receptor gamma-mediated adipogenesis through histone deacetylase recruitment. J. Biol. Chem..

[39] Kamarajugadda S., Becker J.R., Hanse E.A., Mashek D.G., Mashek M.T., Hendrickson A.M., Mullany L.K., Albrecht J.H. (2016). Cyclin D1 represses peroxisome proliferator-activated receptor alpha and inhibits fatty acid oxidation. Oncotarget.

[40] Diehl J.A., Zindy F., Sherr C.J. (1997). Inhibition of cyclin D1 phosphorylation on threonine-286 prevents its rapid degradation via the ubiquitin-proteasome pathway. Genes Dev..

[41] Bieche I., Olivi M., Nogues C., Vidaud M., Lidereau R. (2002). Prognostic value of CCND1 gene status in sporadic breast tumours, as determined by realtime quantitative PCR assays. Br. J. Cancer.

[42] Qie S., Diehl J.A. (2016). Cyclin D1, Cancer Progression and Opportunities in Cancer Treatment. J. Mol. Med. (Berl.).

[43] Guo L., Liu S., Jakulin A., Yilamu D., Wang B., Yan J. (2015). Positive expression of cyclin D1 is an indicator for the evaluation of the prognosis of breast cancer. Int. J. Clin. Exp. Med..

[44] Bosone I., Cavalla P., Chiadò-Piat L., Di Vito N., Schiffer D. (2001). Cyclin D1 expression in normal oligodendroglia and microglia cells: Its use in the differential diagnosis of oligodendrogliomas. Neuropathology.

[45] Lundgren K., Brown M., Pineda S., Cuzick J., Salter J., Zabaglo L., Howell A., Dowsett M., Landberg G., Trans A. (2012). Effects of cyclin D1 gene amplification and protein expression on time to recurrence in postmenopausal breast cancer patients treated with anastrozole or tamoxifen: A TransATAC study. Breast Cancer Res..

[46] Beca F., Pereira M., Cameselle-Teijeiro J.F., Martins D., Schmitt F. (2015). Altered PPP2R2A and Cyclin D1 expression defines a subgroup of aggressive luminal-like breast cancer. BMC Cancer.

[47] Chien H., Cheng S., Liao C., Wang H., Huang S. (2019). Amplification of the EGFR and CCND1 Are Coordinated and Play Important Roles in the Progression of Oral Squamous Cell Carcinomas. Cancers.

[48] Schulze K., Imbeaud S., Letouzé E., Alexandrov L.B., Calderaro J., Rebouissou S., Couchy G., Meiller C., Shinde J., Soysouvanh J. (2015). Exome sequencing of hepatocellular carcinomas identifies new mutational signatures and potential therapeutic targets. Nat. Genet..

[49] Finn R.S., Crown J.P., Lang I., Boer K., Bondarenko I.M., Kulyk S.O., Ettl J., Patel R., Pinter T., Schmidt M. (2015). The cyclin-dependent kinase 4/6 inhibitor palbociclib in combination with letrozole versus letrozole alone as first-line treatment of oestrogen receptor-positive, HER2-negative, advanced breast cancer (PALOMA-1/TRIO-18): A randomised phase 2 study. Lancet Oncol..

[50] Pernas S., Tolaney S.M., Winer E.P., Goel S. (2018). CDK4/6 inhibition in breast cancer: Current practice and future directions. Ther. Adv. Med. Oncol..

[51] Hickey T.E., Robinson J.L., Carroll J.S., Tilley W.D. (2012). Minireview: The androgen receptor in breast tissues: Growth inhibitor, tumor suppressor, oncogene?. J. Mol. Endocrinol..

[52] Lanzino M., De Amicis F., McPhaul M.J., Marsico S., Panno M.L., Andò S. (2005). Endogenous Coactivator ARA70 Interacts with Estrogen Receptor α (ERα) and Modulates the Functional ERα/Androgen Receptor Interplay in MCF-7 Cells. J. Biol. Chem..

[53] Ando S., De Amicis F., Rago V., Carpino A., Maggiolini M., Panno M.L., Lanzino M. (2002). Breast cancer: From estrogen to androgen receptor. Mol. Cell. Endocrinol..

[54] Lanzino M., Sisci D., Morelli C., Garofalo C., Catalano S., Casaburi I., Capparelli C., Giordano C., Giordano F., Maggiolini M. (2010). Inhibition of cyclin D1 expression by androgen receptor in breast cancer cells-identification of a novel androgen response element. Nucleic Acids Res..

[55] Mauro L., Pellegrino M., Giordano F., Ricchio E., Rizza P., De Amicis F., Catalano S., Bonofiglio D., Panno M.L., Ando S. (2015). Estrogen receptor-alpha drives adiponectin effects on cyclin D1 expression in breast cancer cells. FASEB J..

[56] De Amicis F., Chiodo C., Morelli C., Casaburi I., Marsico S., Bruno R., Sisci D., Andò S., Lanzino M. (2019). AIB1 sequestration by androgen receptor inhibits estrogen-dependent cyclin D1 expression in breast cancer cells. Cancer.

[57] Landskroner-Eiger S., Qian B., Muise E.S., Nawrocki A.R., Berger J.P., Fine E.J., Koba W., Deng Y., Pollard J.W., Scherer P.E. (2009). Proangiogenic contribution of adiponectin toward mammary tumor growth in vivo. Clin. Cancer Res..

[58] Bockmeyer C.L., Christgen M., Müller M., Fischer S., Ahrens P., Länger F., Kreipe H., Lehmannet U. (2011). MicroRNA profiles of healthy basal and luminal mammary epithelial cells are distinct and reflected in different breast cancer subtypes. Breast Cancer Res. Treat..

[59] Joyce D.P., Kerin M.J., Dwyer R.M. (2016). Exosome-encapsulated microRNAs as circulating biomarkers for breast cancer. Int. J. Cancer.

[60] He L., Thomson J.M., Hemann M.T., Hernando-Monge E., Mu D., Goodson S., Powers S., Cordon-Cardo C., Lowe S.W., Hannon G.J. (2005). A microRNA polycistron as a potential human oncogene. Nature.

[61] Yu Z., Wang C., Wang M., Li Z., Casimiro M.C., Liu M., Wu K., Whittle J., Ju X., Hyslop T. (2008). A cyclin D1/microRNA 17/20 regulatory feedback loop in control of breast cancer cell proliferation. J. Cell. Biol..

[62] Yu Z., Wang L., Wang C., Ju X., Wang M., Chen K., Loro E., Li Z., Zhang Y., Wu K. (2013). Cyclin D1 induction of Dicer governs microRNA processing and expression in breast cancer. Nat. Commun..

[63] Wang G., Gormley M., Qiao J., Zhao Q., Wang M., Di Sante G., Deng S., Dong L., Pestell T., Ju X. (2018). Cyclin D1-mediated microRNA expression signature predicts breast cancer outcome. Theranostics.

[64] Lü J., Zhao Q., Ding X., Guo Y., Li Y., Xu Z., Li S., Wang Z., Shen L., Chen H. (2020). Cyclin D1 promotes secretion of pro-oncogenic immuno-miRNAs and piRNAs. Clin. Sci..

[65] Patil M.A., Lee S.A., Macias E., Lam E.T., Xu C., Jones K.D., Ho C., Marcelo Rodriguez-Puebla M., Chen X. (2009). Role of Cyclin D1 as a Mediator of c-Met– and β-Catenin–Induced Hepatocarcinogenesis. Cancer Res..

[66] Xia W., Lo C.M., Poon R.Y.C., Tan To Cheung T.T., Chan A.C.Y., Chen L., Yang S., Tsao G.S.W., Wang X.Q. (2017). Smad inhibitor induces CSC differentiation for effective chemosensitization in cyclin D1- and TGF-β/Smad-regulated liver cancer stem cell-like cells. Oncotarget.

[67] Liu H., Fang Y., Wang J., Yuan X., Fan Y., Gao S., Han L., Wang K. (2020). Hypomethylation of the cyclin D1 promoter in hepatitis B virus-associated hepatocellular carcinoma. Medicine.

[68] Orr S.J., Gaymes T., Ladon D., Chronis C., Czepulkowski B., Wang R. (2010). Reducing MCM levels in human primary T cells during the G(0) →4G(1) transition causes genomic instability during the first cell cycle. Oncogene.

[69] Malvezzi M., Carioli G., Rodriguez T., Negri E., La Vecchia C. (2016). Global trends and predictions in ovarian cancer mortality. Ann. Oncol..

[70] Qian W., Dong Y., Yang Y., Liu Z., Feng Y., Ma D., Zhang Z., Wu S. (2014). Gankyrin is frequently overexpressed in cervical high grade disease and is associated with cervical carcinogenesis and metastasis. PLoS ONE.

[71] Chen J., Bai M., Ning C., Xie B., Zhang J., Liao H., Xiong J., Tao X., Yan D., Xi X. (2015). Gankyrin facilitates FSH driven OC cell proliferation through phosphatidylinositol 3-kinases (PI3K)/AKT pathway, the central regulator of OC converging on CD1. Oncogene.

[72] Bell D., Berchuck A., Birrer M., Cancer Genome Atlas Research Network (2011). Integrated genomic analyses of ovarian carcinoma. Nature.

[73] Wang Y., Li W., Wang Z., Ren H., Li Y., Zhang Y., Yang P., Pan S. (2019). Genistein Upregulates Cyclin D1 and CDK4 Expression and Promotes The proliferation of Ovarian Cancer OVCAR-5 Cells.

[74] Bali A., O’Brien P.M., Edwards L.S., Sutherland R.L., Hacker N.F., Henshall S.M. (2004). Cyclin D1, p53, and p21Waf1/Cip1 Expression Is Predictive of Poor Clinical Outcome in Serous Epithelial Ovarian Cancer. Clin. Cancer Res..

[75] Xia B., Yang S., Liu T., Lou G. (2015). miR-211 suppresses epithelial ovarian cance proliferation and cell-cycle progression by targeting Cyclin D1 and CDK6. Mol. Cancer.

[76] Hanken H., Gröbe A., Cachovan G., Smeets R., Simon R., Sauter G., Heiland M., Blessmann M. (2014). CCND1 amplification and cyclin D1 immunohistochemical expression in head and neck squamous cell carcinomas. Clin. Oral Investig..

[77] Albero R., Enjuanes A., Demajo S., Castellano G., Pinyol M., García N., Capdevila C., Clot G., Suárez-Cisneros H., Shimada M. (2018). Cyclin D1 overexpression induces global transcriptional downregulation in lymphoid neoplasms. J. Clin. Investig..

[78] Park Y., Kim S., Lee B., Kim H., Song I., Shin H., Han Y., Chang K., Kim J., Lee D. (2013). Prx I Suppresses K-ras-Driven Lung Tumorigenesis by Opposing Redox-Sensitive ERK/Cyclin D1 Pathway. Antoxid. Redox Signal..

[79] Aquila S., Santoro M., Caputo A., Panno M.L., Pezzi V., De Amicis F. (2020). The Tumor Suppressor PTEN as Molecular Switch Node Regulating Cell Metabolism and Autophagy: Implications in Immune System andTumor Microenvironment. Cells.

[80] Li J., Yin L.L., Su K.L., Zhang G.F., Wang J. (2014). Concomitant depletion of PTEN and p27 and overexpression of cyclin D1 may predict a worse prognosis for patients with post-operative stage II and III colorectal cancer. Oncol. Lett..

[81] Dragoj M., Milosevic Z., Bankovic J., Dinic J., Pesic M., Tanic N., Stankovic T. (2015). Association of CCND1 overexpression with KRAS and PTEN alterations in specific subtypes of non-small cell lung carcinoma and its influence on patients’ outcome. Tumor Biol..

[82] Sun W., Song L., Ai T., Zhang Y., Gao Y., Cui J. (2013). Prognostic value of MET, cyclin D1 and MET gene copy number in non-small cell lung cancer. J. Biomed. Res..

[83] Lawson C.D., Ridley A.J. (2017). Rho GTPase signaling complexes in cell migration and invasion. J. Cell Biol..

[84] Alhaja E., Adan J., Pagan R., Mitjans F., Cascalló M., Rodríguez M., Noé V., Ciudad C.J., Mazo A., Vilaró S. (2004). Anti-migratory and anti-angiogenic effect of p16: A novel localization at membrane ruffles and lamellipodia in endothelial cells. Angiogenesis.

[85] Zhong Z., Yeow W.-S., Zou C., Wassell R., Wang C., Pestell R.G., Quong J.N., Quong A. (2010). Cyclin D1/cyclin-dependent kinase 4 interacts with filamin A and affects the migration and invasion potential of breast cancer cells. Cancer Res..

[86] Fusté N.P., Fernández-Hernández R., Cemeli T., Mirantes C., Pedraza N., Rafel M., Torres-Rosell J., Colomina N., Ferrezuelo F., Dolcet X. (2016). Cytoplasmic cyclin D1 regulates cell invasion and metastasis through the phosphorylation of paxillin. Nat. Commun..

[87] Tang K., Boudreau C.G., Brown C.M., Khadra A. (2018). Paxillin phosphorylation at serine 273 and its effects on Rac, Rho and adhesion dynamics. PLoS Comput. Biol..

[88] Montalto F.I., Giordano F., Chiodo C., Marsico S., Mauro L., Sisci D., Aquila S., Lanzino M., Panno M.L., Andò S. (2019). Progesterone Receptor B signaling Reduces Breast Cancer Cell Aggressiveness: Role of Cyclin-D1/Cdk4 Mediating Paxillin Phosphorylation. Cancers.

[89] Fusté N.P., Castelblanco E., Felip I., Santacana M., Fernández-Hernández R., Gatius S., Pedraza N., Pallarés J., Cemeli T., Valls J. (2016). Characterization of cytoplasmic cyclin D1 as a marker of invasiveness in cancer. Oncotarget.

[90] Jirawatnotai S., Hu Y., Michowski W., Elias J.E., Becks L., Bienvenu F., Zagozdzon A., Goswami T., Wang Y.E., Clark A.B. (2011). A function for cyclin D1 in DNA repair uncovered by protein interactome analyses in human cancers. Nature.

[91] Body S., Esteve-Arenys A., Miloudi H., Recasens-Zorzo C., Tchakarska G., Moros A., Bustany S., Vidal-Crespo A., Rodriguez V., Lavigne R. (2017). Cytoplasmic cyclin D1 controls the migration and invasiveness of mantle lymphoma cells. Sci. Rep..

[92] Liotta L.A., Kohn E.C. (2001). The microenvironment of the tumour-host interface. Nature.

[93] Mishra P.J., Mishra P.J., Humeniuk R., Medina D.J., Alexe G., Mesirov J.P., Ganesan S., Glod J.W., Banerjee D. (2008). Carcinoma-Associated Fibroblast–Like Differentiation of Human Mesenchymal Stem Cells. Cancer Res..

[94] He Y., Franco O.E., Jiang M., Williams K., Harold D., Love H.D., Coleman I.M., Peter S., Nelson P.S., Hayward S.W. (2007). Tissue-Specific Consequences of Cyclin D1 Overexpression in Prostate Cancer Progression. Cancer Res..

[95] Ciznadija D., Liu Y., Pyonteck S.M., Holland E.C., Koff A. (2011). Cyclin D1 and cdk4 mediate development of neurologically destructive oligodendroglioma. Cancer Res..

[96] Pestell T.G., Jiao X., Kumar M., Peck A.R., Prisco M., Deng S., Li Z., Ertel A., Casimiro M.C., Ju X. (2017). Stromal cyclin D1 promotes heterotypic immune signaling and breast cancer growth. Oncotarget.

[97] Chen Y., Huang Y., Gao X., Li Y., Lin J., Chen L., Chang L., Chen G., Guan Y., Pan L.K. (2020). *CCND1* Amplification Contributes to Immunosuppression and Is Associated With a Poor Prognosis to Immune Checkpoint Inhibitors in Solid Tumors. Front. Immunol..

[98] Teo Z.L., Versaci S., Dushyanthen S., Caramia F., Savas P., Mintoff C.P., Zethoven M., Virassamy B., Luen S.J., McArthur G.A. (2017). Combined CDK4/6 and PI3Kα Inhibition Is Synergistic and Immunogenic in Triple-Negative Breast Cancer. Cancer Res..

